# Comparison of the Detection Rate and Specificity of Irregular Red Blood Cell Antibodies Between First-Time Pregnant Women and Women With a History of Multiple Pregnancies Among 18,010 Chinese Women

**DOI:** 10.1155/2024/5539776

**Published:** 2024-05-31

**Authors:** Shujie Wu, Yinglin Wu, Ganping Guo, Rungui Xie, Yuanjun Wu

**Affiliations:** ^1^ Department of Transfusion Medicine Dongguan Maternal and Child Health Hospital, Dongguan, Guangdong 523000, China; ^2^ Prenatal Diagnostic Centre Dongguan Maternal and Child Health Hospital, Dongguan, Guangdong 523000, China

**Keywords:** hemolytic disease of the fetus and newborn (HDFN), hemolytic transfusion reactions (HTRs), irregular red blood cell antibodies, pregnancy

## Abstract

**Background:** There is insufficient evidence to assess the risk of the production of clinically important alloimmune irregular red blood cell (RBC) antibodies in first-time pregnant women.

**Methods:** Using the microcolumn gel antiglobulin method, 18,010 Chinese women with a history of pregnancy and pregnant women were screened for irregular RBC antibodies, and for those with positive test results, antibody specificity was determined. The detection rate and specificity of irregular RBC antibodies in women with a history of multiple pregnancies (two or more) and first-time pregnant women were determined.

**Results:** In addition to 25 patients who passively acquired anti-D antibodies via an intravenous anti-D immunoglobulin injection, irregular RBC antibodies were detected in 121 (0.67%) of the 18,010 women. Irregular RBC antibodies were detected in 93 (0.71%) of the 13,027 women with a history of multiple pregnancies, and antibody specificity was distributed mainly in the Rh, MNSs, Lewis, and Kidd blood group systems; irregular RBC antibodies were detected in 28 (0.56%) of the 4983 first-time pregnant women, and the antibody specificity was distributed mainly in the MNSs, Rh, and Lewis blood group systems. The difference in the percentage of patients with irregular RBC antibodies between the two groups was insignificant (*χ*^2^ = 1.248, *P* > 0.05). Of the 121 women with irregular RBC antibodies, nine had anti-Mur antibodies, and one had anti-Di^a^ antibodies; these antibodies are clinically important but easily missed because the antigenic profile of the reagent RBCs that are commonly used in antibody screens does not include the antigens that are recognized by these antibodies.

**Conclusion:** Irregular RBC antibody detection is clinically important for both pregnant women with a history of multiple pregnancies and first-time pregnant women. Mur and Di^a^ should be included in the antigenic profile of reagent RBCs that are used for performing antibody screens in the Chinese population.

## 1. Introduction

Irregular red blood cell (RBC) antibodies are antibodies against blood group antigens other than ABO antigens and can cause difficulties in blood typing and cross-matching, hemolytic transfusion reactions (HTRs) of varying severity, and hemolytic disease in the fetus and newborn (HDFN) [1, 2]. It is generally accepted that the irregular RBC antibodies produced by “natural immunity” are mainly IgM antibodies, which are not clinically important [3]; irregular RBC antibodies that are clinically important are mainly IgG antibodies, which are the less common type and are produced due to pregnancy, allogeneic blood transfusion, allogeneic tissue and organ transplantation, and other forms of allogeneic immunity [4–6]. It was previously believed that fetomaternal hemorrhage occurs mainly in late pregnancy or during delivery; clinically significant alloimmune irregular RBC antibodies are rarely produced during the first pregnancy without a history of other alloimmunizations; and clinically significant irregular RBC IgG antibodies are produced after the second period of alloimmunization [4, 7]. However, a recent study showed that fetal RBCs can be detected in maternal blood samples at 6–22 weeks of gestation [8], but there is a lack of research data to guide the clinical management of maternal and fetal blood and determine whether fetomaternal hemorrhage can lead to the production of alloimmune irregular RBC antibodies in pregnant women during their current pregnancy.

To understand the risk of the production of irregular RBC antibodies during the current pregnancy as a result of gestational alloimmunization, we performed screens for irregular RBC antibodies in 4983 pregnant women during their late first pregnancy and 13,027 women patients with a history of multiple (two or more) pregnancies, and we compared the differences in irregular RBC antibody positivity and specificity between the two groups; these results will be important for guiding clinical practice in maternal and fetal/neonatal blood management.

## 2. Materials and Methods

### 2.1. Participants

An irregular RBC antibody screening test was performed on 18,010 Chinese women aged 16–40 years who were admitted to the Gynecology and Obstetrics Department of Dongguan Maternal and Child Health Hospital (located in the Pearl River Delta in South China) between January 2021 and February 2023 and likely needed to receive blood transfusion therapy for any reason, including but not limited to anticipated blood loss during delivery. A total of 13,027 women who were currently pregnant or not but who had a history of multiple (two or more) pregnancies and 4983 first-time pregnant women who were in the late stages of pregnancy were included.

### 2.2. Irregular RBC Antibody Screening

With the Aigel 400 fully automatic blood typing instrument (Shenzhen Aikang Bio-technology Co.), a microcolumn gel Coombs card (Diagnostic Grifols, S.A.) was used to perform the irregular RBC antibody screening tests. To minimize the possibility of failing to detect irregular RBC antibodies against relatively low-frequency antigens, especially anti-Mur and anti-Di^a^ antibodies, which are more common and clinically important in the Chinese population than in the European and American populations, the plasma of the patients was reacted with the RBCs of donors who expressed Mur and Di^a^ and had a negative direct antiglobulin test (DAT) result as well as the commercial antibody screening reagent RBCs (Diagnostic Grifols, S.A.), which do not include cells that are positive for Mur and Di^a^. The reaction temperature was 37°C, and the reaction time was 15 min. Agglutination with any of the cells was considered a positive irregular RBC antibody screening test result. A history of nonpregnancy alloimmunization, such as allogeneic blood transfusion, was investigated in the first-time pregnant women with detectable irregular RBC antibodies.

### 2.3. Irregular RBC Antibody Identification

For subjects with positive irregular RBC antibody screening results, microcolumn gel antiglobulin tests were manually performed to determine antibody specificity. Specifically, the plasma of the patient was mixed with each of the panel cells for antibody identification, incubated at 37°C for 30 min, added to a Coombs card, and centrifuged in a special centrifuge. The specificity of irregular RBC antibodies was determined according to the pattern of the agglutination reaction between the panel cells for antibody identification and the plasma of the patient. The panel cells for antibody identification were provided by Guangdong Yingan Biotechnology Co., Ltd. (Guangzhou, China), and the labeled antigens included D, C, E, c, e, M, N, S, s, Mur, P_1_, Le^a^, Le^b^, K, k, Fy^a^, Fy^b^, Jk^a^, Jk^b^, Lu^a^, Lu^b^, and Di^a^.

### 2.4. Statistical Analyses

The data are presented as numbers and frequencies, and the detection rate and specificity of irregular RBC antibodies in women with a history of multiple (two or more) pregnancies and first-time pregnant women were determined separately. SPSS 21.0 statistical software was used, and the difference in the irregular RBC antibody detection rate between the two groups was analyzed using the *χ*^2^ test; *P* < 0.05 was considered to indicate a statistically significant difference.

## 3. Results

### 3.1. Positivity Rate for Irregular RBC Antibodies

Irregular RBC antibodies were detected in 146 of the 18,010 obstetrics and gynecology patients who were likely to require blood transfusions. Twenty-five patients were excluded because they received an intravenous anti-D immunoglobulin injection during pregnancy, which caused anti-D antibody positivity. After the exclusion of these 25 patients, the rate of positivity for irregular RBC antibodies was 0.67% (121/18,010). Among the 4983 first-time pregnant women, irregular RBC antibodies were detected in 31 women, two of whom had a history of allogeneic transfusion and three of whom tested positive for anti-D antibodies because they received an intravenous anti-D immunoglobulin injection during pregnancy; excluding patients who passively acquired anti-D antibodies through an intravenous anti-D immunoglobulin injection, the irregular RBC antibody positivity rate was 0.56% (28/4,983). Among the 13,027 women with a history of multiple pregnancies, irregular RBC antibodies were detected in 115 patients, 22 of whom tested positive for anti-D antibodies because they received an intravenous anti-D immunoglobulin injection during pregnancy; excluding those who passively acquired anti-D antibodies through an intravenous anti-D immunoglobulin injection, the irregular RBC antibody positivity rate was 0.71% (93/13,027), which was not significantly different from the irregular RBC antibody positivity rate in first-time pregnant women (*χ*^2^ = 1.248, *P* > 0.05).

### 3.2. Specificity of Irregular RBC Antibodies

In addition to the passive acquisition of anti-D antibodies through an intravenous anti-D immunoglobulin injection, irregular RBC antibodies were detected in 28 of the 4983 first-time pregnant women, and the distribution of antibody specificity, in descending order, was anti-M, anti-E, anti-Le^a^, anti-c and anti-Mur, anti‐E + anti‐Di^a^, anti-P_1_, and warm autoantibodies. Three patients could not be identified due to limitations in the distribution of cellular antigens in the panel cells for antibody identification. Among the 13,027 women with a history of multiple pregnancies, 93 had irregular RBC antibodies detected, and the distribution of antibody specificity, from most to least, was as follows: anti-E, anti-M, anti-Mur and anti-Le^a^, anti-cE, anti-D, anti-C, anti-Jk^a^ and anti-Jk^b^, anti-e, anti-Ce, anti-Fy^b^, anti-P_1_, and cold autoantibodies. The specificity could not be identified for 12 patients due to limitations in the distribution of cellular antigens in the panel cells for antibody identification. The key differences in the specific distribution of irregular RBC antibodies between the two groups were as follows. Rh blood group system antibodies were significantly more frequently detected in the 13,027 women with a history of multiple pregnancies than in the 4983 first-time pregnant women (*χ*^2^ = 4.968, *P* < 0.05). Among the 13,027 women with a history of multiple pregnancies, Kidd blood group system antibodies were detected in six patients, and Duffy blood group system antibodies were detected in one patient, but antibodies to both blood group systems were not detected in the 4983 first-time pregnant women. The distribution of antibody specificities is shown in Table 1.

## 4. Discussion

The results of this study, which included 18,010 obstetrics and gynecology patients who might require transfusion therapy, showed that except for those with the passive acquisition of anti-D antibodies through intravenous anti-D immunoglobulin injections, the irregular RBC antibody positivity rate among women with a history of multiple (two or more) pregnancies and first-time pregnant women was 0.67%, which was lower than that in previous reports [9–11]. This is mainly because the prevention of alloimmune anti-D antibody production in RhD-negative pregnant women has gradually been emphasized [12, 13]. In recent years, anti-D immunoglobulin has been widely used in RhD-negative pregnant women in areas such as the Pearl River Delta Region of China, reducing the production of alloimmune anti-D antibodies in RhD-negative pregnant women and thus lowering the irregular RBC antibody positivity rate in those with a history of pregnancy.

In addition to the ABO blood group system, antibodies that recognized D, C, c, E, e, M, N, S, s, Fy^a^, Fy^b^, Jk^a^, Jk^b^, K, k, Le^a^, Le^b^, P_1_, Di^a^, and Mur, which are 20 antigens that belong to eight blood group systems (Rh, Kidd, MNS, Duffy, Kell, Lewis, P, and Diego), can cause severe HTRs and HDFN, which are clinically important [14, 15]. Although the use of anti-D immunoglobulin has reduced the production of alloimmune anti-D antibodies in RhD-negative pregnant women, of all irregular RBC antibodies detected in the women with a history of multiple (two or more) pregnancies, antibodies to the Rh blood group system (the most clinically important) still accounted for 47.31% (44/93) of the patients; other clinically important antibodies, such as anti-M, anti-Mur, anti-Jk^a^, anti-Jk^b^, and anti-Fy^b^ antibodies, accounted for 30.11% (28/93) of the patients. The specificity of irregular RBC antibodies could not be determined in 12.90% (12/93) of the patients, and some of these antibodies may be clinically important [16]. The positivity rate and specificity distribution of irregular RBC antibodies among the women with a history of multiple pregnancies in this study support the conclusions of previous studies that irregular RBC antibodies should be tested in patients with a history of pregnancy [17, 18].

There are few studies on the detection rate and antibody-specific distribution of irregular RBC antibodies in first-time pregnant women, and there is insufficient evidence to indicate whether first-time pregnant women should be tested for the presence of irregular RBC antibodies during pregnancy. In this study, the difference in the positivity rate of irregular RBC antibodies between 4983 first-time pregnant women and 13,027 women with a history of multiple pregnancies was insignificant. Among the irregular RBC antibodies detected in the first-time pregnant women, Rh blood group system antibodies accounted for 25% (7/28). Except for one patient with anti-c antibodies and one patient with anti-E and anti-Di^a^ antibodies who had a history of blood transfusion, Rh blood group system antibodies still accounted for 17.86% (5/28). Additionally, 46.43% (13/28) of the patients had anti-M and anti-Mur antibodies, which are clinically important. These findings suggest that first pregnancy-induced alloimmunization can lead to the production of clinically important irregular RBC antibodies in women during their first pregnancy and that screening for irregular RBC antibodies should be performed for first-time pregnant women.

Although our findings suggest the need to test both women with a history of multiple pregnancies and first-time pregnant women for the presence of irregular RBC antibodies, the distribution of certain clinically important antibodies varies between women with a history of multiple pregnancies and first-time pregnant women. The results of this study showed that Rh blood group system antibodies were significantly more frequently detected in women with a history of multiple pregnancies than in first-time pregnant women, and antibodies to both the Kidd and Duffy blood group systems were detected in women with a history of multiple pregnancies but not in first-time pregnant women. This suggests that more attention should be given to the detection of irregular RBC antibodies in women with a history of multiple pregnancies.

In Europe and the United States, the frequency of Mur and Di^a^ expression and the probability of producing anti-Mur and anti-Di^a^ antibodies are very low; therefore, the British Committee for Standards in Hematology (BCSH) and the Food and Drug Administration (FDA) recommend that the antigenic profiles of commercialized antibody screening reagent RBCs do not include Mur and Di^a^ [19, 20]. However, Mur and Di^a^ are relatively common in East Asian populations, and the frequencies of Mur and Di^a^ in the Chinese population are approximately 5.76% and 5.19%, respectively [14, 21–23], with a higher probability of Mur and Di^a^ isoimmunization. The antigenic profiles of antibody screening reagent RBCs have not been established for the Chinese population, and most of the antibody screening reagent RBCs currently used in China do not express the Mur and Di^a^ antigens, which can easily lead to the omission of the detection of anti-Mur and anti-Di^a^ antibodies [24, 25]. In this study, 18,010 obstetrics and gynecology patients had a history of pregnancy; anti-Mur was detected in nine patients, and anti-Di^a^ antibodies were detected in one patient. Both anti-Mur and anti-Di^a^ antibodies can cause severe HDFN and severe HTRs [26–31]. Therefore, antibody screening reagents for RBCs expressing Mur and Di^a^ antigens should be selected for use in irregular RBC antibody screening in China and other regions where Mur and Di^a^ expression is high.

This study has limitations. For instance, although we investigated the transfusion history of first-time pregnant women with irregular RBC antibodies and confirmed that only two patients had a history of blood transfusion, we were still unable to obtain sufficient information to exclude patients with a history of transfusion from all the women with a history of multiple pregnancies and first-time pregnant women; therefore, we were unable to analyze the differences in positivity rates and specificity distributions of irregular RBC antibodies between women with a history of multiple pregnancies and first-time pregnant women after excluding patients with a history of blood transfusion from both groups.

## 5. Conclusion

In summary, this study showed that although the use of anti-D immunoglobulin has resulted in a decreasing trend of irregular RBC antibody positivity in women, the irregular RBC antibody positivity rate in women with a history of pregnancy is still 0.67%, and the specific distribution of antibodies is still dominated by the Rh, MNSs, and Kidd blood group systems, which include clinically important antibodies. During the first pregnancy, irregular RBC antibodies of clinical significance, such as anti-E, anti-Mur, and anti-c antibodies, can be produced, and it is necessary to detect irregular RBC antibodies in first-time pregnant women. Mur and Di^a^ should be included in the antigenic profile of the antibody screening reagent RBCs applied to the Chinese population to avoid the omission of anti-Mur and anti-Di^a^ antibodies, which are two clinically important antibodies.

## Figures and Tables

**Table 1:** Distribution of irregular RBC antibody specificity.

**Specific antibody**		**Multiple pregnancies (** **n** **(%))**	**Primigravida (** **n** **(%))**
Rh (*n* = 50)	Anti-E	28 (30.10)	4 (14.29)
	Anti-D^*^	4 (4.30)	0 (0.00)
	Anti-C	3 (3.22)	0 (0.00)
	Anti-c^**^	0 (0.00)	2 (7.14)
	Anti-e	2 (2.15)	0 (0.00)
	Anti-cE	6 (6.45)	0 (0.00)
	Anti-Ce	1 (1.07)	0 (0.00)

Rh and Diego (*n* = 1)	Anti-E, anti-Di^a^^***^	0 (0.00)	1 (3.57)

MNSs (*n* = 34)	Anti-M	14 (15.05)	11 (39.29)
	Anti-Mur	7 (7.52)	2 (7.14)

Lewis (*n* = 10)	Anti-Le^a^	7 (7.52)	3 (10.71)

Kidd (*n* = 6)	Anti-Jk^a^	3 (3.22)	0 (0.00)
	Anti-Jk^b^	3 (3.22)	0 (0.00)

P (*n* = 2)	Anti-P_1_	1 (1.07)	1 (3.57)

Duffy (*n* = 1)	Anti-Fy^b^	1 (1.07)	0 (0.00)

Other (*n* = 17)	Cold autoantibodies	1 (1.07)	0 (0.00)
	Warm autoantibodies	0 (0.00)	1 (3.57)
	Failure to identify the specificity	12 (12.90)	3 (10.71)

Sum		93 (100.00)	28 (100.00)

^*^A total of 22 patients with a history of multiple pregnancies and three first-time pregnant women in which anti-D antibodies were acquired passively via an intravenous anti-D immunoglobulin injection during pregnancy were not included.

^**^Anti-c was detected in two first-time pregnant women, one of whom had a history of blood transfusion.

^***^Anti-E and anti-Di^a^ antibodies were detected in one first-time pregnant woman with a history of blood transfusion.

## Data Availability

The datasets used and/or analyzed during the current study are available from the corresponding author upon reasonable request.

## Abbreviations

RBC: Red blood cell
HTRs: Hemolytic transfusion reactions
HDFN: Hemolytic disease in the fetus and newborn
DAT: Direct antiglobulin test
BCSH: British Committee for Standards in Hematology
FDA: Food and Drug Administration.
